# The Impact of Insurance Status on the Outcomes after Aneurysmal Subarachnoid Hemorrhage

**DOI:** 10.1371/journal.pone.0078047

**Published:** 2013-10-29

**Authors:** Pui Man Rosalind Lai, Hormuzdiyar Dasenbrock, Ning Lin, Rose Du

**Affiliations:** Department of Neurosurgery, Brigham and Women’s Hospital and Harvard Medical School, Boston, Massachusetts, United States of America; D’or Institute of Research and Education, Brazil

## Abstract

Investigation into the association of insurance status with the outcomes of patients undergoing neurosurgical intervention has been limited: this is the first nationwide study to analyze the impact of primary payer on the outcomes of patients with aneurysmal subarachnoid hemorrhage who underwent endovascular coiling or microsurgical clipping. The Nationwide Inpatient Sample (2001–2010) was utilized to identify patients; those with both an ICD-9 diagnosis codes for subarachnoid hemorrhage and a procedure code for aneurysm repair (either via an endovascular or surgical approach) were included. Hierarchical multivariate regression analyses were utilized to evaluate the impact of primary payer on in-hospital mortality, hospital discharge disposition, and length of hospital stay with hospital as the random effects variable. Models were adjusted for patient age, sex, race, comorbidities, socioeconomic status, hospital region, location (urban versus rural), and teaching status, procedural volume, year of admission, and the proportion of patients who underwent ventriculostomy. Subsequent models were also adjusted for time to aneurysm repair and time to ventriculostomy; subgroup analyses evaluated for those who underwent endovascular and surgical procedures separately. 15,557 hospitalizations were included. In the initial model, the adjusted odds of in-hospital mortality were higher for Medicare (OR 1.23, p<0.001), Medicaid (OR 1.23, p<0.001), and uninsured patients (OR 1.49, p<0.001) compared to those with private insurance. After also adjusting for timing of intervention, Medicaid and uninsured patients had a reduced odds of non-routine discharge (OR 0.75, p<0.001 and OR 0.42, p<0.001) despite longer hospital stays (by 8.35 days, p<0.001 and 2.45 days, p = 0.005). Variations in outcomes by primary payer–including in-hospital post-procedural mortality–were more pronounced for patients of all insurance types who underwent microsurgical clipping. The observed differences by primary payer are likely multifactorial, attributable to varied socioeconomic factors and the complexities of the American healthcare delivery system.

## Introduction

Legislation impacting healthcare insurance has remained at the forefront of politics, policy, and media in the United States for more than half a century. Medicare and Medicaid enrollments have been steadily increasing for several decades: in the most recent census, the two programs were the primary payer for more than 108 million Americans. Despite this expansion of coverage, disparities based on insurance status have been increasingly recognized for patients with varied medical and surgical conditions. Medicare and Medicaid patients undergoing a range of operations have been found to have greater mortality and morbidity, an observation partially attributable to complexities in the American healthcare delivery system and socioeconomic factors [1], [2], [3], [4], [5]. Previous studies have suggested that non-private insurance status is associated with decreased utilization of diagnostic testing and intervention in the emergency department and poorer post-surgical outcomes after operations performed emergently [6], [7], [8]. Although investigation into differences in outcomes by insurance status for patients undergoing neurosurgical intervention has been limited, a recent retrospective institutional study found that patients with public or no insurance were more likely to sustain a complication after craniotomy [9]. However, no nationwide study to date has evaluated the impact of primary payer on the outcomes after surgical or endovascular securing of ruptured cerebral aneurysms.

Intracranial aneurysms affect approximately 2–4% of the population [10], [11], [12]. Despite advances in surgical techniques and neurological intensive care, mortality and morbidity remain high for patients with ruptured cerebral aneurysms. The standard management for aneurysmal subarachnoid hemorrhage is to perform a ventriculostomy if there is evidence of hydrocephalus followed by aneurysm repair, either via neurosurgical clipping or endovascular coiling. Patients who undergo early intervention may have superior outcomes, including reduced mortality and less morbidity [13].

Prior studies have found hospitalization costs for both endovascular and surgical treatment of ruptured aneurysms to be higher than Medicare payments [14]. However, no analysis to date has evaluated the relationship between insurance status and outcomes after aneurysmal subarachnoid hemorrhage. This is the first nationwide study to investigate the impact of primary payer status on the in-hospital outcomes–mortality, length of stay, and discharge disposition–after surgical or endovascular treatment of ruptured intracranial aneurysms.

## Methods

### Data Source

Data were extracted from the Nationwide Inpatient Sample (NIS, Healthcare Cost and Utilization Project, Agency Healthcare Research and Quality) from January 1, 2001 to December 31, 2010. The NIS is the largest all-payer inpatient database in the US, consisting of approximately 8 million annual hospitalizations. The database contains all discharges from sampled hospitals and is an approximately 20% stratified sample of American non-federal hospitals. The NIS contains information about diagnoses, procedures, patient demographics, payment sources, length of stay, discharge status, and hospital characteristics to allow for analysis of national trends in health care (http://www.hcup-us.ahrq.gov).

### Inclusion Criteria and Outcome Measures

Patients were included if they had an *International Classification of Diseases, 9^th^ Revision, Clinical Modification (ICD-9-CM)* diagnosis code for subarachnoid hemorrhage (SAH, ICM-9-CM 430) or intracerebral hemorrhage (ICH, ICM-9-CM 431); those with ICH were included as patients with specific aneurysm locations may present after rupture primarily with ICH rather than SAH. However, patients were only included if they had at least one procedural code for aneurysm repair, by “clipping of aneurysm” (ICM-9-CM 39.51), “endovascular repair or occlusion” (ICM-9-CM 39.72), or “other repair of aneurysm” (ICM-9-CM 39.79). Patients were identified by primary payer status, which is directly encoded in the NIS, and stratified into four groups: Medicare, Medicaid, private insurance, and uninsured. Patients were classified as uninsured if their insurance status was coded as self-pay or no charge.

In-hospital mortality, hospital discharge disposition, and length of hospital stay were selected as outcome measures. Discharge disposition is classified by the NIS as 1) routine (to home), 2) transfer to a short-term hospital, 3) other transfer, including to a skilled nursing facility, 4) home health care, 5) against medical advice, 6) died, and 7) unknown. A non-routine discharge was defined as any category other than the first, and only calculated for patients discharged alive.

### Statistical Analysis

Analyses were performed using STATA 12.0 (StataCorp LP, College Station, Texas) using a hierarchical regression model with hospital as the random effect to account for the complex sampling of NIS. Probability values were considered statistically significant if *p*<0.05.

Univariate analysis of two-group mean comparison test and F-test were used to compare study population demographics. The Wilcoxon rank sum test and Kruskal-Wallis test were used for non-normal distributions, identified by the Shapiro-Wilk normality test.

Multivariate hierarchical logistic and linear regression analyses were performed to evaluate the outcomes of patients undergoing repair of ruptured cerebral aneurysms by primary payer. Potential confounding variables included as covariates were patient demographics (patient age, sex, race, comorbidities, and the median household income for the patient’s ZIP code), hospital demographics (teaching status, region, location, and procedural volume), year of admission, differences in the severity of presentation (the performance of a ventriculostomy and the presence of intracerebral hemorrhage), and variations in management (treatment modality, time to aneurysm repair, and time to ventriculostomy). Patient age, sex, and comorbidities (using the Elixhauser *et al*. categories of comorbid disease [15]) are directly coded in the NIS.

Hospitals are coded in the NIS by teaching status, location (urban versus rural), and region. A hospital is classified as a teaching hospital if it has an AMA-approved residency program, is a member of the Council of Teaching Hospital (COTH), or has a ratio of full-time equivalent interns and residents to beds of 0.25 or higher. Urban or rural location is determined by Core Based Statistical Area (CBSA) codes. Hospital region is classified by the NIS as Northeast, Midwest, South, and West. Hospital volume of cerebral aneurysm repair was calculated by counting the total number of endovascular coiling or microsurgical clipping procedures for ruptured aneurysms over the number of years the hospital was included in the dataset. Year of admission was included to account for temporal changes in insurance policies and health care delivery.

Ventriculostomy was used as a surrogate for severity of neurological deficit upon presentation–as this procedure is traditionally reserved for patients with a Hunt-Hess grade of 3 or greater–and identified by procedural codes (ICM-9-CM 02.2). Likewise, the presence of intracerebral hemorrhage was also utilized as a potential maker of severity of presentation, as these patients by definition have a higher Fisher grade. The proportion of patients treated via microsurgical clipping was included as a covariate to adjust for differences in treatment modality.

Time to aneurysm repair and ventriculostomy were calculated by identifying the number of days from admission to procedural intervention, which is directly encoded in the NIS. Finally, to analyze the degree to which any differences which may be present based on insurance status are impacted by the procedural approach utilized to secure the aneurysm, subgroup analyses were performed separately for patients who underwent surgical clipping and endovascular coiling.

## Results

### Study Population

17,559 patients with aneurysmal subarachnoid hemorrhage underwent microsurgical clipping or endovascular coiling between 2001 and 2010; the demographics of the study population are presented in **Table 1**. The majority of patients were covered by private insurance (50.6%), followed by Medicare (23.3%), Medicaid (14.7%), and no insurance (11.4%). Medicare patients were older (mean age 69, *p*<0.0001), while Medicaid (mean age 46, *p*<0.0001) and uninsured patients (mean age 47, *p*<0.0001) were younger, compared to those with private insurance (mean age 50). The Medicare group had a higher proportion of females (74% versus 67% of privately insured patients, *p*<0.0001), while the uninsured group had a lower proportion (62%, *p*<0.0001).

**Table 1:** The demographics of patients with aneurysmal subarachnoid hemorrhage who underwent surgical clipping or endovascular coiling, by insurance status.

	Private	Medicare		Medicaid		Uninsured		*F*
			*p* *		*p* *		*p* *	
No. of patients	8883	4096		2578		2002		
**Age** (mean±SD)	50±11	69±11	**<0.0001**	46±13	**<0.0001**	47±11	**<0.0001**	**<0.0001**
**Female** (%)	67	74	**<0.0001**	67	0.7680	62	**<0.0001**	**<0.0001**
**Race** (%)			**0.0020**		**<0.0001**		**<0.0001**	**<0.0001**
White	68	72		40		50		
Black	14	12		25		22		
Hispanic	10	9.2		25		20		
Asian/Pacific Islander	4.5	4.0		3.8		3.4		
Native American	0.3	0.27		0.7		0.2		
Other	3.7	3.0		5.2		4.8		
**Number of Comorbidities** **(median±range)** +	1 (0–8)	2 (0–9)	**<0.0001**	2 (0–9)	**<0.0001**	1 (0–8)	**0.0009**	**0.0001**
**Median Household** **Income (%)**			**<0.0001**		**<0.0001**		**<0.0001**	**<0.0001**
< $25,000	18	25		37		34		
$25,000–34,999	23	27		27		28		
$35,000–44,999	26	23		22		23		
>$44,999	33	24		14		16		
**Microsurgical Clipping (%)**	60	52	**<0.0001**	60	0.5014	63	**0.0498**	**<0.0001**
**Ventriculostomy (%)**	26	34	**<0.0001**	34	**<0.0001**	20	**0.0002**	**<0.0001**
**Intracerebral** **Hemorrhage (%)**	8.8	11	**0.0060**	9.7	0.2142	6.8	**0.0094**	**0.0002**

* P values are for comparison with private insurance.

+ For non-normal distributions, Wilcoxon rank sum and Kruskal-Wallis tests were used.

We investigated the baseline characteristics of the hospitals in which the patient population was treated (**Table 2**). A greater proportion of Medicaid (67%, *p* = 0.0065) and uninsured patients (66%, *p* = 0.0199) were treated at a teaching hospital compared to those with private insurance (59%). Hospital location and region did not differ significantly by primary payer. Those with Medicare underwent intervention at hospitals with higher annual volume of ruptured aneurysms (*p* = 0.0075). Time to aneurysm treatment was significantly longer for Medicare and Medicaid patients (*p*<0.0001). Time to ventriculostomy was not different among the four insurance groups.

**Table 2:** Characteristics of the hospitals treating patients with aneurysmal subarachnoid hemorrhage via surgical clipping or endovascular coiling, by insurance status.

	Private	Medicare		Medicaid		Uninsured		*F*
			*p* *		*p* *		*p* *	
**Teaching hospital (%)**	59	60	0.5920	67	**0.0065**	66	**0.0199**	**0.0131**
**Rural location (%)**	3.3	3.0	0.7950	1.8	0.1464	2.7	0.6146	0.5340
**Hospital region (%)**			0.9994		0.6112		0.6298	0.8517
Northeast	17	17		16		15		
Midwest	22	22		22		23		
South	36	37		36		43		
West	24	24		26		18		
Annual Aneurysm ProcedureVolume (median+range)+	27(0.14–134)	30.5(0.14–134)	0.0075	25.8(0.25–134)	0.2128	25.8(0.25–134)	0.7574	
Time to Aneurysm Repair, days(median+range)+	1 (0–69)	1 (0–50)	<0.0001	1 (0–106)	<0.0001	1 (0–40)	0.1138	**<0.001**
Time to Ventriculostomy, days(median+range)+	0 (0–52)	0 (0–62)	0.2955	0 (0–77)	0.4034	0 (0–42)	0.6828	0.209

* P values are for comparison with private insurance.

+ For non-normal distributions, Wilcoxon rank sum and Kruskal-Wallis test are used for nonparametric alternative to two-sample t-test and ANOVA respectively.

### Univariate Analysis

First, univariate analysis was performed to evaluate the unadjusted difference in outcomes by primary payer. In-hospital mortality was higher for patients with Medicare (20%, *p*<0.0001), Medicaid (12%, *p* = 0.0299) and no insurance (14%, *p* = 0.0008) compared to patients with private insurance (11%). The proportion of patients with non-routine discharge was higher for Medicare patients (83%, *p*<0.0001), not different for Medicaid patients (59%, *p* = 0.06), and lower for those without insurance (43%, *p*<0.0001) compared to the privately insured. Medicaid patients had the longest unadjusted length of hospital stay (25±23 days, *p*<0.001), followed by those with Medicare (21±14 days, *p*<0.001), no insurance (19±15, *p* = 0.211), and private insurance (19±13 days).

### Multivariate Analyses

Subsequently, hierarchical multivariate regression models were constructed to evaluate differences in outcomes by insurance status after adjusting for several potential confounding variables (**Table 3**). The initial multivariate regression model included patient and hospital demographics, year of admission, and severity of presentation as independent variables. A second multivariate model was also constructed including all of the variables in the first model as well as timing of intervention–time to aneurysm repair and time to ventriculostomy.

**Table 3:** Hierarchical analysis* evaluating the impact of primary payer status on the outcomes after aneurysmal subarachnoid hemorrhage, utilizing patients with private insurance as the reference.

Outcome	Medicare		Medicaid		Uninsured	
	OR [95% CI]	*p*	OR [95% CI]	*p*	OR [95% CI]	*p*
**In-hospital Mortality**	1.23 [1.14, 1.34]	<0.001	1.23 [1.14, 1.34]	<0.001	1.49 [1.36, 1.63]	<0.001
**Non-routine Discharge**	1.62 [1.48, 1.76]	<0.001	1.08 [1.002, 1.16]	0.044	0.53 [0.49, 0.58]	<0.001
	**Coef. [95% CI]**	***p***	**Coef. [95% CI]**	***p***	**Coef. [95% CI]**	***p***
**Length of Stay**	0.80 [−0.046, 1.65]	0.064	6.00 [4.76, 7.23]	<0.001	0.64 [−0.22, 1.49]	0.144

* The independent variables included as covariates in regression analyses were patient age, sex, race, comorbidities, median household income of the patient’s zip code, hospital region, hospital location, teaching status, procedural volume, the presence of intracerebral hemorrhage, the performance of a ventriculostomy, year of admission, and aneurysm treatment modality (clipping or coiling).

In the initial model, the adjusted odds of in-hospital mortality were significantly higher for Medicare (OR 1.23, p<0.001), Medicaid (OR 1.23, *p*<0.001) and uninsured patients (OR 1.49, *p*<0.001) compared to patients with private insurance. Additionally, Medicare and Medicaid beneficiaries had higher adjusted odds of non-routine discharges (OR 1.62, p<0.001 and OR 1.08, p = 0.044) while uninsured patients had lower adjusted odds of a non-routine discharge. Length of stay was longer for Medicaid patients (by 6 days, p<0.001) but not significant for those with Medicare or uninsured status. In the second model, when adjusted for timing of intervention, both Medicaid (OR 0.75, p<0.001) and uninsured patients (OR 0.42, p<0.001 had reduced non-routine discharges despite longer length of stay (by 8.35 days, p<0.001 for Medicaid and by 2.45, p = 0.05 for uninsured) (**Table 3**).

To determine whether differences in outcome measures were associated with the procedural approach utilized for securing the aneurysm, subgroup analyses were subsequently performed separately for patients who underwent microsurgical clipping and endovascular coiling (**Tables 4** **and** **5**). Compared with those with private insurance, Medicare patients who underwent surgical clipping had a higher adjusted odds of in-hospital mortality (OR 1.36, p<0.001) and non-routine discharges (OR 1.50, p<0.001) but not difference in length of hospital stay. Medicaid patients had a higher in-hospital mortality (OR 1.18, p = 0.025) and no difference in non-routine discharges despite an increased in hospital length of stay (by 7.63 days, p<0.001). Uninsured patients had a lower adjusted odds of a non-routine discharge (OR 0.30, p<0.001) and longer length of stay (by 2.93 days, p = 0.018). Patients who underwent endovascular coiling had no difference in mortality by insurance status.

**Table 4:** Subgroup analyses investigating the impact of primary payer on the outcomes after aneurysmal subarachnoid hemorrhage in patients who underwent microsurgical clipping.

	PrivateInsurance	Medicare			Medicaid			Uninsured		
	%	%	OR[95% CI]	*p*	%	OR[95% CI]	*p*	%	OR[95% CI]	*p*
**In-hospital** **Mortality**	10.5	19.3	1.36[1.16, 1.58]	<0.001	12.3	1.18[1.02, 1.36]	0.025	13.4	1.09[0.88, 1.36]	0.438
**Non-routine** **Discharge**	59.2	84.4	1.50[1.32, 1.71]	*<0.001*	57.8	1.03[0.93, 1.14]	0.632	42.5	0.30[0.25, 0.35]	<0.001
	**Days**	**Days**	**Coef.** **[95% CI]**	***p***	**Days**	**Coef.** **[95% CI]**	***p***	**Days**	**Coef.** **[95% CI]**	***p***
**Length** **of Stay**	19±13	23±15	0.13[−1.30, 1.56]	0.857	26±24	7.63[5.38, 9.87]	<0.001	20±15	2.93[0.49, 5.36]	0.018

* The independent variables included as covariates in regression analyses were patient age, sex, race, comorbidities, median household income of the patient’s zip code, hospital region, hospital location, teaching status, procedural volume, the presence of intracerebral hemorrhage, the performance of a ventriculostomy, and year of admission.

**Table 5:** Subgroup analyses investigating the impact of primary payer on the outcomes after aneurysmal subarachnoid hemorrhage in patients who underwent endovascular coiling.

	Private Insurance	Medicare			Medicaid			Uninsured		
	%	%	OR[95% CI]	*p*	%	OR[95% CI]	*p*	%	OR[95% CI]	*p*
**In-hospital** **Mortality**	11.5	21.6	0.85[0.58, 1.26]	0.418	12.5	1.31[0.95, 1.81]	0.104	13.8	1.26[0.75, 2.10]	0.381
**Non-routine** **Discharge**	54.0	80.9	2.06[1.45, 2.93]	<0.001	60.5	1.76[1.29, 2.40]	<0.001	43.8	0.39[0.26, 0.58]	<0.001
	**Days**	**Days**	**Coef.** **[95% CI]**	***p***	**Days**	**Coef.** **[95% CI]**	***p***	**Days**	**Coef.** **[95% CI]**	***p***
**Length of** **Stay**	18±13	20±13	−3.08[−7.76, 1.60]	0.197	24±20	12.1[4.85, 19.4]	0.001	18±14	8.49[−1.41, 18.4]	0.093

* The independent variables included as covariates in regression analyses were patient age, sex, race, comorbidities, median household income of the patient’s zip code, hospital region, hospital location, teaching status, procedural volume, the presence of intracerebral hemorrhage, the performance of a ventriculostomy, and year of admission.

## Discussion

The association between socioeconomic disadvantage and poor health has been well-established. Even in the cases of emergency care where access is not determined by insurance status, difference in care can still occur with respect to the utilization of diagnostic testing and intervention [16], [17], [18]. The factor of insurance status, which is often treated as a binary parameter and a surrogate for socioeconomic status, has recently been considered an independent variable in public-health analyses [19]. Numerous reports have suggested that patients with public or no insurance have inferior outcomes after certain medical and surgical treatments compared to patients covered by private insurance [1], [3], [5], [8], [9], [20], [21], [22], [23], [24], [25], [26], [27], including those who underwent neurosurgical procedures [28], [29]. However, no study to date has analyzed the impact of insurance status on the outcomes after aneurysmal subarachnoid hemorrhage: we report the first nationwide study evaluating if differential outcomes exist after microsurgical clipping or endovascular coiling for ruptured intracranial aneurysms by payer status.

In this study, 17,559 patients with aneurysmal subarachnoid hemorrhage from across the United States who presented over a ten year period were evaluated. After adjusting for many confounding variables–including patient and hospital demographics–patients with Medicaid, Medicare and uninsured patients had higher adjusted odds of in-hospital mortality compared to those with private insurance. Length of hospital stay was significantly longer by 6 days for Medicaid patients. After adjusting for time to treatment, both Medicaid and uninsured patients had reduced non-routine discharges despite increased length of stay. When subgroup analyses evaluated patients undergoing clipping and coiling separately, differences in outcomes based upon insurance status, including in-hospital mortality, were more profound for those who underwent clipping compared to patients treated via endovascular coiling.

Time to ventriculostomy and aneurysm repair have been associated with clinical outcomes in patients with ruptured intracranial aneurysms [13], but their usage in previous database analyses has been limited. In this study, time to aneurysm treatment was significantly longer for Medicare patients compared to those with private insurance. When the timing of intervention was added to multivariate regression models, the impact of insurance status on non-routine discharges and length of stay became more pronounced.

The reason for the differences in outcomes and timing of intervention by primary payer seen in this study may be multifactorial. Prior studies have suggested that disparities based on insurance status are largely attributable to underlying societal variations and the structure of the healthcare delivery system in the United States rather than individual provider or hospital bias. It has been hypothesized that inferior outcomes for patients with suboptimal insurance undergoing surgical procedures may be partially attributable to three factors–access to high-quality care, comorbidities, and acuity of presentation [30]. Those with government-sponsored or no insurance are known to have prohibitive language and transportation barriers to receiving quality care. Individuals with private insurance may possess better access to primary care, have greater health-care literacy, and additional pre- and post-operative support [9], [19]. Patients with limited access to primary care may have poorly controlled comorbidities, which are known to negatively impact surgical outcomes, including those with subarachnoid hemorrhage [31], and may delay procedural intervention. While twenty-nine comorbidities were included as covariates in multivariate analyses, this may not fully account for differences in severity of the diseases. Moreover, patients with limited access to primary care and preventive medicine may have reduced screening for diseases and referral to specialists–including cerebrovascular neurosurgeons–potentially decreasing the likelihood of undergoing elective securing of the cerebral aneurysm.

Notably, Medicaid and uninsured patients were associated in this study with a 25% and 58% lower adjusted odds of a non-routine hospital discharge, despite having significantly longer hospitalizations (by 8.35 and 2.45 days, respectively). These observations are consistent with prior studies which have found restricted access to rehabilitation services for uninsured patients after traumatic injury [32]. Although governmental programs such as Medicaid are designed to provide coverage for acute conditions, there may be variability in extension to include rehabilitation. This may explain why Medicaid patients were less likely to have non-routine discharges and had longer hospital stays: those that were medically eligible for rehabilitation may have remained in the hospital until they were ready to be discharged home.

The limitations of this study merit closer evaluation. Analysis of the NIS is retrospective, inherently restricting the ability to deduce causal relationships. There is also limited specific clinical information in the NIS that are important predictors of clinical outcome; therefore, multivariate analyses could not include data on the neurological examination upon presentation, Hunt-Hess and Fisher Grade, size and location of the aneurysm, re-bleeding rates, or vasospasm. However, we included the presence of ventriculostomy procedure in our multivariate analysis as a surrogate for severity of presentation. Coding inaccuracies are a potential concern for any study based on ICM-9-CM identifiers; however, there is no reason to suspect that coding errors would preferentially impact patients of a particular primary payer. Furthermore, the mortality rates in this study only represent patients who underwent procedural treatment for ruptured aneurysms and do not include patients who died before intervention. This may bias towards better outcomes, as those with decreased access to healthcare may have a higher chance of dying prior to receiving treatment.

Nonetheless, the NIS has many unique advantages that make it well-suited to evaluate the impact of insurance status on surgical outcomes. The NIS is the largest all-payer database in the United States, providing a broader perspective than state-wide or single-payer datasets. The NIS includes data on many potential confounding variables–including patient and hospital demographics–that were utilized as covariates in multivariate analyses. The large size and well-established nationwide perspective of the NIS have rendered it a standard dataset for large-sample analyses on health care utilization and outcomes in the United States. Further research, particularly with data collected in a prospective fashion, may provide additional insight into strategies to reduce differential outcomes based on primary payer for patients undergoing procedural aneurysm repair after aneurysmal subarachnoid hemorrhage.
